# The willingness and perceptions of Surinamese individuals in the Netherlands on living tissue donation: A qualitative and exploratory study

**DOI:** 10.1371/journal.pone.0338125

**Published:** 2025-12-15

**Authors:** Charifa Zemouri, Assia Nait Kassi, Muriel Sinselmeijer, Bert Elbertse, Yvonne Mulder, Sara Jobse, Ilyaz Nasrullah, Jennifer Jubitane, Shelaika Hoogdorp, Eva-Maria Merz

**Affiliations:** 1 Zemouri et al., Amsterdam, the Netherlands; 2 MentalEdGroup, Abu Dhabi, United Arab Emirates; 3 Department of Research and Laboratory Services, Sanquin Blood Supply Foundation, Amsterdam, the Netherlands; 4 Matchis Foundation, Leiden, the Netherlands; 5 Dutch Transplant Foundation, Leiden, the Netherlands; 6 Rumified, Rotterdam, the Netherlands; 7 Department of Social Studies, Fontys Hogeschool, Eindhoven, the Netherlands; 8 Huisartsen Spoedpost Paramaribo, Paramaribo, Suriname; 9 Donor Behaviour Group, Sanquin Research Amsterdam, Amsterdam, the Netherlands; 10 Department of Sociology, Vrije Universiteit Amsterdam, Amsterdam, the Netherlands; University of Amsterdam, NETHERLANDS, KINGDOM OF THE

## Abstract

**Background:**

The Surinamese population in the Netherlands is an ethnically diverse group with a specific need for transfusion or transplantation due to a higher prevalence of diseases like beta-thalassaemia and sickle cell disease. This study explored the willingness of Surinamese individuals in the Netherlands to donate blood, stem cell, and live organ donation, and preferred information dissemination methods.

**Methods:**

A sequential, qualitative, exploratory study was conducted using an online questionnaire and a focus group to examine the willingness to donate living tissue. Participants were Surinamese individuals aged 18–55 residing in the Netherlands. The questionnaires were inductively thematically analysed. The results led to in-depth questioning and discussion among the focus group, which consisted of four men and four women. The session was recorded and transcribed verbatim for thematic analysis.

**Results:**

We identified ten themes across tissue types: 1) awareness of needs; 2) information and knowledge; 3) donation process; 4) cues to action; 5) attitude; 6) religion; 7) health challenges; 8) fear; 9) social cohesion and solidarity; 10) relationship with recipient. Willingness to donate blood was high, but they faced barriers, including registration challenges and limited access. Stem cell donation was seen as invasive. Living organ donation was considered only for emotionally close recipients. Participants were unaware of shortages and the importance of ethnic matching. They called for inclusive campaigns reflecting broader ethnic diversity, not just Suriname. Future strategies should simplify access to information, registration, and donation processes.

**Conclusion:**

Most participants were unfamiliar with living donations, and perceived barriers were key reasons for not donating. Emotional bonds and awareness of ethnic matching and shortages motivated willingness. Participants stressed the need for tailored, ethnic-specific campaigns to address barriers and misconceptions, emphasise ethnic matching, and highlight reliance on their communities for successful living donation.

## Introduction

Ethnic matching between donors and patients is crucial for successful transplantation and transfusion outcomes [1,2]. Compatibility between donors and recipient lowers rejection risk, reduces complications, and improves survival rates. Many European and North American societies are becoming increasingly diverse due to demographic changes. Yet, donor populations in these countries do not reflect this shift. In the Netherlands, over 25% of the population has a non-Dutch ethnic background [3–6]. Therefore, a diverse human tissue donor population is necessary to provide living donations of stem cells, blood products, and organs for patient care. As of 2024, the Netherlands has a population of approximately 18 million, of which 450,000 blood donors, 415,000 registered stem cell donors (about 2.5% of the Dutch population) and 533 living organ donors. The Dutch stem cell registry estimates that 95% of donors are of North-West European descent. Similarly, blood donors from minority ethnic groups are underrepresented, posing challenges for tissue transplantation as ethnic matching is vital for health outcomes of the patient. Research indicates that individuals of Moroccan, Turkish, and Surinamese descent are less likely to register as organ donors and are underrepresented in living kidney donations [2,6]. The exact estimate of the ethnic diversity of the Dutch donor pool is unknown because ethnicity is not registered in donation institutes in the Netherlands.

Shortage of suitable products for patients from non-North-West European descent is particularly evident in Rotterdam, the most diverse city in the Netherlands [2]. In Rotterdam, 44% of the persons on the waiting list for a kidney transplantation are of non-European descent. Yet, only 15% of the potential donors in Rotterdam match this demographic [2]. The lack of representation, especially among those of African descent [7], lowers the likelihood of finding compatible donors, increases health disparities like higher morbidity and mortality when a patient needs donation and burdens the Dutch healthcare demand for tissue transplantations [8,9].

The Surinamese population in the Netherlands, which has settled in the Netherlands over multiple generations since colonial times and after Suriname’s independence, faces significant challenges in finding compatible donors. This difficulty stems from their ethnically diverse origins, which include backgrounds from Sub-Saharan Africa, the Indian Peninsula, Indonesia, and China [10]. Additionally, many Surinamese individuals are of mixed ethnicity, increasing genetic diversity and complicating donor matching [11]. A particular concern is the African-descended subgroup within the Surinamese population, which has a higher prevalence of blood-related diseases such as sickle cell disease and beta-thalassemia [9,12]. This group requires a consistent blood supply from a genetically diverse donor pool. Complete HLA-matched blood donors are crucial to prevent alloimmunization and to meet the needs of patients who have developed complications from prior transfusions [9,13]. Moreover, the unique HLA-typing within this diverse community also challenges finding exact HLA matches for stem cell and organ transplantation [14].

Recruiting ethnically diverse living donors, particularly from the Surinamese community, is crucial to meet the demand for blood, stem cell, and organ (kidney and liver) donations, and to increase the availability of HLA-matched donors in the Netherlands [9]. Current efforts must become more effective in reaching, recruiting, and retaining potential donors [2,13]. In response, the Dutch Ministry of Health commissioned this study in collaboration with the Dutch National Blood supply (Sanquin), the foundation for stem cell registry in the Netherlands (Matchis Foundation), and the Dutch Transplantation Society (Nederlandse Transplantatie Stichting, NTS). This qualitative study aims to assess views of Surinamese individuals on living donation, their willingness to donate, and their preferred methods for receiving information to inform and develop evidence-based recruitment campaigns.

## Methods

### Study design

This study utilised an online questionnaire followed by a focus group, employing a qualitative and explorative design that allowed for a comprehensive analysis of participants’ opinions and insights on living donation [15]. The manuscript was completed and assessed with the consolidated criteria for reporting qualitative research (COREQ-32), a checklist for qualitative research [16].

In the Netherlands, the Surinamese community does not have a formalized leadership structure, largely due to its substantial ethnic diversity. As such, community-level consent was not applicable. Public and patient involvement (PPI) was applied instead and enhanced the study’s setup and execution by forming an advisory group consisting of three professionals who identify as Surinamese and actively contributed to this study [17]. C.Z. purposively selected advisory group members for their active community participation, diverse Surinamese ethnic backgrounds, and professional expertise. The group (n = 3) consisted of a 35-year-old female midwife of Surinamese-Creole ethnicity who works in a clinic in Paramaribo, Suriname and Amsterdam, the Netherlands (S.H.), a 39-year-old female diversity and inclusion specialist of Surinamese-mixed ethnicity (J.J.), and a 37-year-old male digital and artificial intelligence specialist with a focus on public information of Surinamese-Hindustani ethnicity (I.N.). The advisory groups supported the creation of culturally appropriate and easy-to-understand online questionnaires, distributed them within their networks, and aided in recruiting focus group participants. After collecting the questionnaire data, they participated in data interpretation and validation. Their insights were then used to co-develop detailed focus group questions.

### Study setting, population and recruitment

This study was conducted in preparation for a nationwide targeted donor recruitment and information campaign by three Dutch donor organisations: Sanquin, Matchis, and NTS. The Netherlands has over 350,000 inhabitants of Surinamese descent, including first-, second-, and third-generation immigrants. They were invited to participate in this study by completing a questionnaire and/or joining a focus group. The study included individuals aged 18–55 (the eligibility age for stem cell donation) who identified as Surinamese, were living in the Netherlands, and specified their Surinamese ethnic background and were proficient in Dutch to complete a survey and express themselves adequately. In this study, ethnicity refers to participants’ self-identified cultural and ancestral affiliation within the Surinamese population, encompassing diverse subgroups such as Creole (African descent), Hindustani (Indian descent), Javanese (Indonesian descent), and mixed backgrounds. Due to this self-identification aspect of ethnicity, different categories of ethnicity might be used. The focus group participants were purposefully selected to achieve a balanced representation of gender, age, ethnicity, and educational background. Participants were asked about their attitudes towards living donations during the recruitment process. Participants with positive, negative and ambivalent attitudes toward living donations were invited to participate. Potential participants with a medical profession were excluded to prevent potential influence on group dynamics. The focus group was held in Dutch in the community cultural centre in Amsterdam New-West.

### The online questionnaire

Data from study participants was collected through an online questionnaire created in Qualtrics, from 20/01/2024–02/02/2024. The questionnaire was initially tested within the researchers’ network and by the advisory group to ensure clarity and relevance. The questionnaire began with a cover page explaining the study’s purpose, identifying the funding institute and the associated donor institutions, and clarifying the concept of “living donation” to prevent confusion with post-mortem donation. Participants were required to give consent on this page before proceeding further. The questionnaire included a combination of closed demographic questions and open-ended questions aimed at capturing participants’ experiences and opinions in detail (see Supplementary File 1, S1, The online questionnaire). Questions were tailored to align with the Dutch donation registration system. Blood and organ donation items were retrospective, given that registered blood donors have usually already donated and that there is no national registry for living organ donors. For stem cell donation, the focus was on registration status, as only a small proportion of registered individuals are ever matched and called for donation. Our primary interest was in those who are registered to evaluate diversity within the donor registry. Given the high ethnic and religious diversity within the Surinamese population, questions about self-reported ethnicity and religion were included to analyse potential diversity in responses relative to participants’ backgrounds.

We aimed to recruit adults who identify as Surinamese using convenience and snowball sampling, inviting them to complete an online questionnaire and/or join a focus group. To reach the target population effectively, the research team, advisory group, and their networks distributed the questionnaire link and an accompanying message via WhatsApp and personal messaging on social media platforms like X, Instagram, and LinkedIn. Participants were also encouraged to share the link within their networks to expand outreach. Invitations for focus groups were shared using similar methods.

### Focus group

The focus group participants were recruited from 02/02/2024–28/02/2024 and one focus group (N = 8) on 21/03/2024 to gain in-depth insights, perspectives, and opinions, complementing the questionnaire data. The results from the questionnaire were used to develop probing questions and topics for the focus groups in collaboration with the advisory group. C.Z., a scientific researcher with Ph.D., facilitated the discussion, asked questions, guided participant interactions and took field notes. C.Z. had no relationships with any of the participants. Y.M., B.E., and S.J. supported C.Z. for additional in-depth questioning. The session began with introductions and discussed the willingness to donate blood, stem cells, and organs during life. The session took two hours, and data saturation (defined as the point where no new themes or insights emerged in participant responses [18]) was achieved when all the topics were discussed, the focus group participants had fully elaborated on their views, and no more questions for clarification arose. The discussion was recorded using a Philips voice tracer DVT2050, saved as an MP3 file, and securely stored. The recordings were transcribed verbatim using Amber Script and manually corrected by C.Z. for accuracy. The focus group participants received financial compensation of 50 Euros for their participation.

### Data analysis

The questionnaires remained online until data saturation was reached. Incomplete questionnaires, such as those without answers to open-ended questions or containing responses like ‘none’ or ‘not applicable,’ were excluded from the analysis. Closed questions were analysed using descriptive statistics, while free-text responses and focus group input were subjected to thematic analysis following the six-step plan of Kiger and Varpio [19]. This process involved familiarisation with the data, coding key segments, identifying and refining themes, and defining and naming themes. The questionnaire responses and focus group transcripts were anonymised and analysed inductively, with themes organised into broader categories addressing blood, stem cells, and organs. Participant quotes were selected to illustrate key themes and translated into English for reporting by C.Z. and validated by I.N., J.J., and S.H. Thematic analysis was not conducted on information needs due to limited generated data, as responses were brief, repetitive, or lacked variation. Instead, the data was summarised narratively to ensure accurate representation and inclusion in the analysis.

The analysis and coding were conducted by C.Z. and reviewed by A.N.K., I.N., J.J, and S.H, with any discrepancies resolved through consensus.

### Researcher reflectivity and positionality

I.N., J.J., and S.H. are professionals of Surinamese descent in the Netherlands and Suriname who utilised their expertise, cultural knowledge, and experience to contribute to data gathering and data interpretation. J.J. had a strong sense of language and community communication in her role as a diversity and communication specialist and researcher. C.Z., conducted the focus group discussions with support from Y.M., B.E., and S.J., who are affiliated with a donor registry or collection institution. C.Z. and A.N.K., both women of Moroccan descent who were born and raised in the Netherlands’ multicultural environment, led the data analysis.

The combination of the above-mentioned authors, combined with their extensive experience and training in qualitative research, particularly with ethnic minority groups, brought valuable insights. While our cultural and professional proximity facilitated access and trust, it may also have shaped what participants chose to share and how we interpreted their narratives. For example, shared minority experiences may have made certain nuances more visible, while institutional affiliations or assumptions of shared understanding could have limited others.

### Statement of ethics

#### Study approval statement.

The study was commissioned by the Dutch Ministry of Health, Welfare and Sport. Ethical review was sought from the non-WMO (Medical Research Involving Human Subjects Act, Netherlands) Committee of the Medical Ethics Review Committee (METC) of Amsterdam University Medical Centers. On 04/01/2024, the Committee formally confirmed that the study did not require approval under the WMO regulations (METC reference number 2023.0949). The waiver, provided in written form in both Dutch and English, was communicated by Prof. Dr. J.A.M. van der Post, Chair of the Committee.

#### Consent to participate statement.

The questionnaire participants ticked a consent box before proceeding. The focus group participants were informed in writing about the study and were required to sign a written informed consent form in person prior to participating in the study. Everyone had the liberty to stop the questionnaire any time or withdraw from the focus group.

## Results

### Questionnaire output

Data saturation was reached after 100 responses; data from all 121 participants were included as they had already been collected upon identifying saturation. Approximately 30 questionnaires had to be excluded throughout the data collection because participants were >55 years old or left the questionnaire blank. The participant characteristics of the online questionnaire are reported in Table 1, and those of the focus group in Table 2.

**Table 1:** Questionnaire response overview and characteristics.

Characteristics		
Gender (self-identified)	Female	86 (71.1%)
	Male	33 (27.3%)
	Prefer not to say	1 (0.8%)
	Other	1 (0.8%)
Age (in years)	Mean	41.12
	Standard deviation	9.16
	Range	18–55
Ethnicity (self-identified)	Hindustani	52 (43.0%)
	Mixed	33 (27.3%)
	Creole	28 (23.1%)
	Javanese	7 (5.8%)
	Marron	1 (0.8%)
	Chinese	0 (0.0%)
Religion/faith	None	46 (38.0%)
	Hinduism	33 (27.3%)
	Christianity	24 (19.8%)
	Islam	7 (5.7%)
	Missing answer	7 (5.7%)
	Afro-Surinamese	1 (0.8%)
	Kejawen	1 (0.8%)
	Agnostic	1 (0.8%)
	Omnism	1 (0.8%)
Have you donated blood?	Yes	18 (14.6%)
	No	103 (83.7%)
	I do not know	0 (0.0%)
Are you registered as a stem cell donor?	Yes	14 (11.6%)
	No	93 (76.8%)
	I do not know	14 (11.6%)
Have you ever donated an organ/part of an organ?	Yes	0 (0%)
	No	121 (100%)
**Questionnaire response rate**		
Which reasons do you have for (not) donating blood?		100%
Which reasons do you have for (not) donating stem cells?		99.8%
Which reasons do you have for (not) donating organs?		99.8%
Which information do you need about blood donation?		48.7%
Which information do you need about stem cell donation?		54.4%
Which information do you need about organ living donation?		41.4%
How would you like to be informed?		91.2%

**Table 2:** Focus group participant characteristics.

Participant ID	Gender	Age	Ethnicity (self identified)	Educational level	Work setting	Donor status
V1-C	Woman	39	Creole-Portuguese	Vocational tertiary education	Education	Not a donor
V2-S	Woman	43	Creole	Vocational education	Public service	Donated blood in the past
V3-F	Woman	19	Creole-Nigerian	Vocational tertiary education	Student	Not a donor
V4-Z	Woman	25	Hindustani	Bachelor’s	Student	Not a donor
M1-R	Man	45	Creole	Higher vocational education	Arts and culture	Not a donor
M2-K	Man	33	Creole	Vocational tertiary education	Finance	Donated blood in the past
M3-D	Man	31	Javanese-Chinese-Creole	Master’s	Government	Blood donor
M4-J*	Man	29	Marron-Creole-Mix	Vocational education	Unknown	Unknown

*This participant was invited to join the focus group and excused himself.

**Participant coding:** V = female (*vrouw* in Dutch); M = male (*man* in Dutch). The number reflects the participant’s sequence of speaking within their gender group. The final letter corresponds to the initial of their first name.

### Themes

Ten key themes emerged from the thematic analysis of the written responses. Given the interconnected nature and interpretation of the data, some responses could be classified under multiple themes, resulting in potential overlap. However, our thematic analysis intentionally separates these categories to capture the nuanced ways these barriers manifest and influence different aspects of donation behaviour. By maintaining these distinct categories, we provide a more comprehensive understanding of how various barriers interact with specific stages of the donation decision-making process. Table 3 presents these themes, their conceptualisation, and frequency of occurrence for each tissue type.

**Table 3:** Overview of overarching themes per donation type.

#	Theme	Conceptualization	Blood	Stem cells	Organ
1	Awareness of need	Recognition of the necessity for living donation due to shortages and its importance for patient care.	Yes	Yes	No
2	Information and knowledge	**Top down**: information and knowledge shared from the donation organizations. **Bottom up**: knowledge gaps from the person.	Yes	Yes	Yes
3	Donation process	Obstacles concerning the donation process split into three subthemes: **Registration** (e.g., registration, data handling). **Medical process** (e.g., medical check-up, tissue extraction). **Emotional process** (e.g., emotions linked to the donation process).	Yes	Yes	Yes
4	Cues to action	Stimulus that can prompt action: donation, self-study, reflection.	Yes	Yes	Yes
5	Attitude	The attitude of the person towards donation, this may be negative, neutral, ambivalent, or positive. Trust and distrusts are also aspects included as part of an attitude.	Yes	Yes	Yes
6	Religion	Reasons for donation driven from a religious perspective or motivation. **Barrier**: When a person perceives donation as unlawful by their religion. **Facilitator**: When a person perceives donation as a lawful by their religion.	Yes	Yes	Yes
7	Health challenges	Health issues that are prevalent or may be perceived as challenge and may prevent a person from donation.	Yes	No	No
8	Fear	Experienced fear from the person concerning to donation.	Yes	Yes	Yes
9	Social cohesion and solidarity	Donating can be divided into two main subthemes: **Altruism**: Helping others without expecting anything in return. **Reciprocity**: Assisting others with the expectation of receiving help in return or giving back after having been helped. These capture motivations rooted in broader community values, such as a sense of duty or altruism toward the collective.	Yes	Yes	No
10	Relationship with recipient	The participants’ relationship with a potential recipient. This could be friendship, kinship, romantic or non-existing. In contrast to the previous theme, this theme focuses on the individual relationship a participant may have with a recipient.	No	Yes	Yes

#### Awareness of needs.

This theme highlights the participants’ identified recognition and awareness of the importance of living donations. The questionnaire responses indicated that some respondents knew there was a need for blood and stem cell donations. They recognised the importance of a diverse donor pool. Some respondents already knew of the demand for stem cell donors in their community and registered themselves as donors. However, the focus group participants were unaware of the importance and necessity of diversity in living donors and the necessity of ethnic matching. Once informed during the focus group, their reported willingness to register as donor increased due to the awareness that successful donor matches often require donors from the same ethnicity.

*“When you hear that the blood of a white Dutch man would not match with my father or I, then it [the need to donate] comes closer”. Focus group participant M1-R.*

#### Information and knowledge.

This theme focuses on information and knowledge gaps related to living donation. Questionnaire participants reported that their main concern for abstaining from blood donation was the lack of information and knowledge, although they were somewhat familiar with the concept of blood donation. However, they were less familiar with stem cell and living organ donation. Some responses in the questionnaire indicated that respondents needed to familiarise themselves with the criteria for donation and their eligibility to make an informed decision about whether or not donating living tissue was something they wanted to do.

The focus group participants were unfamiliar with the shortages of living donors and the need for ethnicity-based matching. They responded that this matter is not a topic of discussion in their communities. Raising this topic and spreading this information may increase the willingness of their communities to register as living donors.

“*I am insufficiently informed.” Questionnaire participant, woman, 38 years, Creole ethnicity.*

#### Donation process.

This theme highlights the organisational, practical and procedural aspects of the donation process. The questionnaire identified key barriers to living donation within the donation process, including inconvenience, lack of time, unfamiliarity with the procedure, and the perceived hassle of registration. Concerns about possible bone marrow punctures, invasive procedures, and sequelae were deterring registrations for stem cell donations.

The focus group participants were also unfamiliar with the registration and medical process linked to stem cell and living organ donation. Emotional concerns, such as the fear of living with one kidney, the possibility of caring for their households, and the impact on work and quality of life, were voiced. Some thought registration meant immediate donation and were deterred by the perceived intensity and pain of the procedure. They expressed a need for detailed information on the registration process, the likelihood of being called for donation, the voluntary nature of each step and donating only if matched, believing this would increase willingness to participate. They also suggested locating donation centres closer to the community could ease the process. Furthermore, the group feared that donated blood might be sold for financial gain and expressed concerns about profits related to their donation.

*“Hassle. I think it will be painful, and the risks are unclear. What if I get physical damage? Will the insurance cover me?” Questionnaire participant, woman, 37 years, Hindustani ethnicity.*

#### Cues to action.

This theme explores motivational triggers or the lack thereof. While logistical challenges may act as a barrier, this theme also captures the absence of proactive outreach as missed opportunities to engage potential donors. Most questionnaire respondents indicated they needed prompts, meaning reminders or cues, to donate blood or stem cells. Those hesitant about blood donation were often unaware of the opportunity, lacked time, or had previously attempted to donate but faced obstacles or challenges that prevented them from completing the process. Some had never considered living organ donation or were undecided. The focus group emphasised the need for triggers to encourage blood donation and suggested bringing donation centres closer to the community to facilitate walk-in donations. Participants felt Sanquin lacked visibility, and existing posters did not inspire them to learn more or register as donors. The focus group also noted a need for similar prompts and a lack of cues to action for stem cell and living organ donation.

“*I have never been asked to be a stem cell donor”. Questionnaire participant, man, 33 years, mixed ethnicity.*

#### Attitude.

This theme encompasses broader emotional and cognitive perspectives, such as distrust in donation institutions or cultural stigmas, which reflects a general attitude rather than a procedural issue. The questionnaire revealed varied attitudes towards living donation. Some participants viewed blood donation negatively, citing oddness, religious prohibitions, a sense of obligation or had attitude of distrust towards the donation banks. Others were positive, seeing it as easy and helpful. Stem cell donation received mixed responses: negative views included distrust, unwillingness to donate to strangers, and resistance due to others’ negative experiences, while positive views focused on helping others. Some had neutral reasons, such as lacking a specific motive to be a donor. Attitudes towards living organ donation ranged from objections to surgery and objections against “cutting in a healthy body” to a willingness to donate during life or post-mortem, with some having no specific reason to donate at all.

The Surinamese study population’s attitudes towards blood donation varied strongly due to diversity in cultures, practices, religion and generational differences. Ambivalence towards stem cell donation stemmed from a lack of knowledge and familiarity, and attitudes towards living organ donation were influenced by the relationship with the potential recipient.

“*I don’t want to be cut in my body”. Questionnaire participant, woman, 37 years, Creole ethnicity.*

#### Religion.

Some questionnaire participants reported that their religion influenced their willingness to donate. Some refused living organ donation due to religious prohibitions (Christianity), one person reported that their religion allowed it (Islam), and the other said that it depended on the situation (Christianity). The focus group participants discussed that the Surinamese people are very diverse in culture, ethnicity, and religion, and their denomination within a church may play a role in their willingness to be a living donor. In some churches, blood donation is rarely discussed, and some even consider it taboo. Some of the mentioned examples were beliefs about blood and organs being sacred, or that donation interferes with bodily integrity.

Churches and mosques were reported to influence opinions about donations. Additionally, intergenerational differences in perspectives existed within the various religious subcultures, which may result in younger generations having different views on donation than older generations.

“*If the Lord Jesus permits, let His will be done”. Questionnaire participant, woman, 39 years, Creole ethnicity.*

#### Health challenges.

The questionnaire identified chronic conditions, frail health, hypotension, infectious diseases, blood disorders, and deficiencies as (perceived) common health barriers to living donations. The focus group participants added additional mental health barriers, noting psychological resistance and care-avoidant behaviour. They discussed how some people, particularly from the older generation, are less likely to seek medical help, often turning first to religious, spiritual, or cultural practices. This hesitancy to engage with formal medical care can directly impact their willingness or ability to participate in living donations. Several participants mentioned that some individuals are first advised to pray or take a “*dresie*” (a mix of Surinamese herbs) before consulting a doctor. This practice, although culturally accepted as a healing method, could possibly delay diagnosis and treatment of underlying health conditions, which are critical for determining eligibility for living donation. As a result, these delays may reduce an individual’s readiness or suitability to participate as a living donor.

*“It’s cultural, like if you are sick, you should take this ‘dresie.’ You shouldn’t take paracetamol; you should take ‘dresie’”. Focus group participant M3-D.*

#### Fear.

Fear was a significant reason for abstaining from living donation and registration. For blood donation, fear centred on blood and needles. Fear for stem cell donation were linked to the bone marrow puncture and potential physical consequences. The fear of living with one kidney and undergoing surgery was reported for kidney donation. The focus group participants extensively elaborated on their communities’ perceived fear of donation. They discussed a widespread fear towards healthcare, seeking healthcare and inflicting unnecessary pain on oneself. One participant (M3-D) explained that the ‘*why should I’* question is often posed by persons concerning their willingness.

*“In our language, we have a saying ‘Blacka man no lob skin atti’, which means the black man does not like pain”. Focus group participant M1-R.*

#### Social cohesion and solidarity.

The primary motivations for social cohesion and solidarity included helping others, serving the community, reciprocating, and expressing gratitude. Some questionnaire responses indicated a conditional approach to blood and stem cell donation, with participants preferring to donate to known recipients or only to family and friends. Those considering living organ donation cited solidarity, helping others, and reciprocity as key reasons, viewing saving lives as a crucial donation aspect. The focus group emphasised that Sanquin should highlight the need for diverse blood donations within the Surinamese community, and that successful blood matching often required donors of the same ethnic background as recipients to stimulate social cohesion and collaboration within their group. For Surinamese people, family and community are decisive motivating factors, as they place high value on supporting those within their cultural group. This is particularly important when recognising the need for ethnic matching in living donations, where donors and recipients from the same ethnic background are more likely to have compatible tissue types, increasing the success rate of transplants and reducing the risk of complications.

While participants emphasized both community solidarity and personal relationships, we chose to distinguish these themes to capture the spectrum of motivations. Solidarity reflects broader communal values, while relationships focus on specific interpersonal connections. However, we acknowledge the conceptual overlap with the next theme.

“*Out of solidarity, I hope that if I need an organ, others will be willing to donate it, too.” Questionnaire participant, man, 37 years, Creole ethnicity.*

#### Relationship with recipient.

Respondents to the questionnaire reported that their relationship with the recipient is decisive for living organ donation. Willingness to donate was present when it concerned a first-degree family member such as a child, sibling, or people close to them. The responses also revealed the unwillingness to donate to strangers or persons with whom they do not have emotional ties. Similarly, the focus group participants indicated a willingness to donate an organ to their children, mothers, family members, or friends with whom they have strong ties and whom they wish to help and save. The group was unwilling to donate part of an organ to individuals with whom they do not have a strong bond, even if itis a relative or to strangers, similar to the questionnaire data. The focus group questioned why they should undergo surgery on a healthy body for someone else while they must live with the consequences. Nevertheless, they were willing to face these consequences for their loved ones.

*“Yes, I could really do it (organ donation) for my sister, but my mother wouldn’t do it for her sister. I would also do it for my mother.” Focus group participant V3-F.*

#### Campaigns and communication.

Due to limited questionnaire responses on information needs, only focus group insights were reported and summarised. Participants felt that current campaigns were ineffective and suggested a varied approach. Combining online campaigns with direct, on-site engagements and interactive opportunities, such as booths at festivals or mobile donation units in neighbourhoods or at public transport stations, was preferred. The group stressed that campaigns should be directly linked to actions like registration or accessing additional information.

Communication should be carried out in collaboration with community organisations and individuals, including patients, experts with personal experience and professionals. The focus group noted that the Surinamese community is highly fragmented, requiring different approaches based on generation, religion, and ethnicity. Each group had its church or mosque, followed its denomination, and had its radio programs. Younger people tend to use Instagram, TikTok and podcasts for information, while older individuals prefer Facebook and local Surinamese radio programs.

The focus group participants emphasised the need for more information on living donation, especially pressing shortages and ethnic matching. They highlighted that campaigns should focus on ethnic matching and the Surinamese community’s diverse African and Asian roots, rather than on Suriname as a country. Featuring diverse individuals and stories would ensure representation and highlight the importance of unique matches.

The participants suggested that the topic would be better addressed by groups or individuals who resemble them, for example, through popular podcasts, which discuss various topics or campaigns in which the community can see themselves represented. The group expressed concerns that well-known Surinamese figures do not represent all community groups and that using celebrities could dilute the message. However, Surinamese footballers could serve as role models for youth.

They recommended using popular podcasts and relatable personal stories to make the message more tangible and urgent, such as knowing the recipient or receiving a video message to highlight the impact of their donation.

## Discussion

This sequential qualitative study explored Surinamese individuals’ views on living donation, revealing key barriers such as unawareness about shortages, lack of knowledge, fear of pain and donation-related complications. Understanding the need for living donations, ethnic matching, and increasing knowledge were facilitating factors to remove barriers, resolve misconceptions and urge the need within this community. Participants preferred direct community outreach from the donor institutions with tailored on- and offline campaigns, including persons they can relate to whilst focusing on ethnicity and diversity rather than Suriname as a country due to the community’s high ethnic diversity.

Reasons for abstaining from donation were fear of physical consequences, lack of knowledge, and other perceived barriers. The reasons for abstaining from blood donation align with previous studies and have not really changed over time [20–25]. Participants were less willing to donate stem cells due to unfamiliarity with and knowledge about stem cells and ethnicity-based matching. Living organ donation willingness was strongly tied to the recipients’ relationship, with fears of pain and risks being less significant when donating to a close relative. However, fear remained a dominant barrier to altruistic donations, consistent with previous studies [14,26,27]. Besides fear, distrust towards donor and collection institutions, authorities and healthcare avoidance were also reported as reasons to distance from living donation. A qualitative Netherlands-based study showed that persons with a migration background sometimes experience racism and discrimination in Dutch healthcare, which could contribute to fear of healthcare or healthcare avoidance [28]. Distrust due to negative experiences or socio-political contexts has been extensively studied as a significant barrier in healthcare and recruitment of ethnic minorities in Western countries [7,29–31].

In a previous Dutch study among Dutch persons with Ghanaian and Surinamese-African backgrounds, findings suggested that emotional triggers can enhance decision-making toward donation [20], supported by our current study. The emotional connection with the recipient, an emotional trigger to the need for donors and a sense of community have been studied before as aspects to be targeted in increasing the willingness to donate [32]. Reasons such as helping others, awareness of shortage, and reciprocity were the main reasons for considering living donation. Solidarity, alongside reciprocity and awareness of shortages based on ethnic matching, may help convince people to register as donors and eventually donate [33–35]. As our data revealed, participants’ awareness that they must rely on persons of the same ethnic background increased the sense of emergency to contribute to the donor pool.

This study highlights the need to address the underrepresentation of ethnic minority donors to motivate people to contribute to sufficient blood supply [31,36]. A survey of potential African-American stem cell donors suggested that educational components of recruitment programs should address the most common barriers before addressing the drivers, effectively increasing donor numbers [36]. In the same study, most participants needed to know that ethnicity-based matching was necessary for successful transfusions. This was the same in the current study, and has been addressed as vital information for recruitment campaigns and donor recruitment efforts [37]. Ethnic group-specific recruitment and information campaigns that emphasise ethnic matching and community reliance are necessary to achieve a diverse donor pool [13,31,37]. The Surinamese communities could be reached through varied and interactive approaches, including on-site contact, online campaigns and direct community outreach.

However, recruitment campaigns must consider varied strategies for different generations and diverse community cultures, religions, and information needs. Future studies should explore the effectiveness of tailored, ethnic-based campaigns in increasing donor registration and retention although monitoring ethnic impact may be challenging as ethnicity is not measured in Dutch donor registries.

### Strength and limitation

This study demonstrates several strengths. First, the sequential qualitative design enabled a comprehensive approach to data collection, combining the broad insights gained from an online questionnaire with the depth provided by focus group discussions. This allowed the researchers to explore general trends and more nuanced perspectives within the Surinamese community. The study integrated PPI, where community representatives actively shaped the research and interpreted the findings. This involvement ensured that the survey was culturally relevant and that the insights aligned closely with the population’s lived experiences [35,38]. Furthermore, the study offers practical, targeted insights for future recruitment campaigns, which can be used to address the underrepresentation of ethnic minorities in Dutch donor registries.

The study’s main limitation was the potential sampling bias among the questionnaire participants and focus group members. The first indication of this bias was the relatively high percentage of respondents who reported being blood or stem cell donors. Additionally, individuals who were not interested in the topic or had negative attitudes towards tissue donation were less likely to participate in the study, resulting in the absence of their perspectives. This bias was evident in the data due to the lack of responses reflecting negative attitudes. Given the female-skewed sample, interpretations of attitudes and experiences should be made with caution. Additionally, as our recruitment used convenience and snowball sampling with decentralized onward sharing, the total number approached could not be ascertained; therefore, a conventional response rate and non-response analysis were not possible, and selection bias cannot be excluded.

The questionnaire and focus group were conducted in Dutch, which may have excluded individuals with lower Dutch proficiency or digital literacy, thus missing perspectives from a broader target population. Th underrepresentation of participants with lower educational backgrounds may have constrained the range of perspectives. Future research should include greater educational diversity to capture broader experiences. Although the study aimed to represent the diverse Surinamese population in the Netherlands, some ethnic subgroups (e.g., Marron, Chinese, Javanese) were underrepresented in the sample. Consequently, the discussion leader actively inquired about the participants’ family and social circle perceptions to gather insights into the social norms surrounding living donations, especially since social norms are pivotal in perceiving the living donation subject [39]. Because focus groups were held in Amsterdam, participation from Surinamese-origin individuals in other cities may have been reduced, introducing geographic selection bias and limiting transferability (e.g., to Rotterdam or The Hague). While the Moroccan backgrounds of C.Z. and A.N.K. were not expected to influence data interpretation, their familiarity with the Surinamese community may have contributed to a heightened cultural awareness during the analysis with support of J.J., I.N. and S.H. The other authors (Y.M., B.E., S.J., M.S.) provided technical expertise that likely shaped the study’s direction.

## Conclusion

Surinamese individuals have a diverse and mixed ethnic background and are underrepresented in the Dutch donor registries. This shortage leads to ethnic health disparities and challenges in meeting donor demands based on ethnic matching for HLA typing. Participants of this study cited unfamiliarity with and perceived barriers to the various types of donations as the main reasons for abstaining from living donation. However, the emotional bond and relationship with potential recipients, along with awareness of ethnic matching and ethnic-based shortages, were significant motivators for donation.

To recruit and retain more donors, a tailored ethnic-specific campaign is needed. This campaign should address donation barriers and misconceptions, emphasise the importance of ethnic matching, and highlight the reliance on individuals from the same community for successful living donation. The campaign should evoke an emotional response to the shortages to increase the willingness to register as living donors and ultimately donate when matches are found. Future studies should assess the success of recruiting a more diverse donor pool through improved recruitment campaigns.

## Supporting information

S1 File The online questionnaire. The original questions in Dutch are reported, along with their translations into English.
(DOCX)

S2 File Inclusivity in research questionnaire. (DOCX)

S3 File Qualitative Research Checklist. (PDF)
