# Supplementary material for: The willingness and perceptions of Surinamese individuals in the Netherlands on living tissue donation: A qualitative and exploratory study
Source: PLoS One. 2025 Dec 15;20(12):e0338125. doi: 10.1371/journal.pone.0338125 (PMC12704896; doi:10.1371/journal.pone.0338125)
Supplement: S1 File — The original questions in Dutch are reported, along with their translations into English. (DOCX) [file pone.0338125.s001.docx]

# The online questionnaire.

The original questions in Dutch are reported with the translations to English.

| Part of the questionnaire | Question | Subject | Outcome |
| --- | --- | --- | --- |
| 1: Demografie  *Demography* | 1 | Hoe oud bent u?  *What is your age?* | Number |
|  | 2 | Hoe identificeert u zich?  *How do you identify?* | 1. Man *Man* 2. Vrouw *Woman* 3. Dat zeg ik liever niet *Prefer not to say* 4. Of: (vrij vak) *Or (free space)* |
|  | 3 | Wat is uw etniciteit?  *What is your ethnicity?* | 1. Surinaams-Creeols *Surinamese-Creole* 2. Surinaams-Hindoestaans *Surinamese-Hindustan* 3. Surinaams-Chinees *Surinamese-Chinese* 4. Surinaams-Javaans *Surinamese-Javanese* 5. Surinaams-Maron *Surinamese-Maron* 6. Surinaams-gemengd *Surinamese-Mixed* |
|  | 4 | Belijdt u een geloof?  *Do you practice a belief?* | 1. Ja *Yes* 2. Nee *No* |
| 2: Donorstatus  *Donor status* | 1 | Heeft u ooit bloed gedoneerd?  *Have you ever donated blood?* | 1. Ja *Yes* 2. Nee *No* 3. Weet ik niet *I don’t know* |
|  | 2 | Staat u ingeschreven als stamceldonor?  *Are you registered as stem cell donor?* | 1. Ja *Yes* 2. Nee *No* 3. Weet ik niet *I don’t know* |
|  | 3 | Heeft u ooit een orgaan of stuk van uw orgaan gedoneerd?  *Have you ever donated an organ or piece of your organ?* | 1. Ja *Yes* 2. Nee *No* |
| 3: Bereidheid  *Willingness* | 1 | Welke redenen heeft u om wel of niet bloed te doneren?  *What are your reasons to donate blood?* | |
|  | 2 | Welke redenen heeft u om wel of niet stamcellen te doneren?  *What are your reasons to donate stem cells?* | |
|  | 3 | Welke redenen heeft u om wel of niet tijdens uw leven organen zoals een stuk van uw lever of een nier te doneren?  *What are your reasons to donate organs such as a piece of your liver or your kidney during your life?* | |
|  | 4 | Welke informatie heeft u nodig over:  *What information do you need about:*   1. Bloeddonatie *Blood donation* 2. Stamceldonatie *Stem cell donation* 3. Orgaandonatie tijdens het leven *Organ donation during life* | |
|  | 5 | Hoe wilt u informatie over donatie krijgen? Denk hierbij aan informatiebronnen als familie, huisarts, (sociale) media en alles wat verder bij u opkomt.  *How would you like to receive information about donation? Think about information sources such as family, general practitioner, (social) media and everything else that pops your mind.* | |
